# Vegetable Tannin as a Sustainable UV Stabilizer for Polyurethane Foams

**DOI:** 10.3390/polym11030480

**Published:** 2019-03-12

**Authors:** Maria Oliviero, Mariamelia Stanzione, Marco D’Auria, Luigi Sorrentino, Salvatore Iannace, Letizia Verdolotti

**Affiliations:** 1 Institute of Polymers, Composites and Biomaterials, National Research Council, P.le E. Fermi 1, 80055 Portici (NA), Italy; maria.oliviero@unina.it (M.O.); mariamelia.stanzione@gmail.com (M.S.); marco.dauria@unina.it (M.D.); lverdolo@unina.it (L.V.); 2 Institute for Macromolecular Studies, National Research Council, v. Corti 12, 20133 Milano, Italy; iannace@unina.it

**Keywords:** polyurethane foams, tannin, degradation, mechanical properties, UV radiation

## Abstract

A vegetable tannin, a flavonoid-type natural polyphenolic compound, was used to promote the stabilization of polyurethane foams against UV radiation. Several polyurethane foams were synthesized by using an isocyanate, and a mixture of ethoxylated cocoalkyl amine and vegetable tannin. The content of vegetable tannin was varied from 0 to 40 wt %. The effects of tannin and water (used as a blowing agent) on the foaming kinetics and cellular morphology of foams were investigated. Samples were subjected to accelerated weathering under UV radiation for 3 to 24 h, and FTIR and DMA analyses were conducted to assess the performance change. The former analysis revealed a strong inhibiting effect of tannin on urethane linkage degradation during the UV treatment. The mechanical properties were significantly affected by the addition of tannin. The capability of the foams to withstand UV radiation was dependent on the amount of tannin. At tannin contents higher than 20%, the decrease in mechanical properties under UV irradiation was almost avoided.

## 1. Introduction

**Figure 1:**
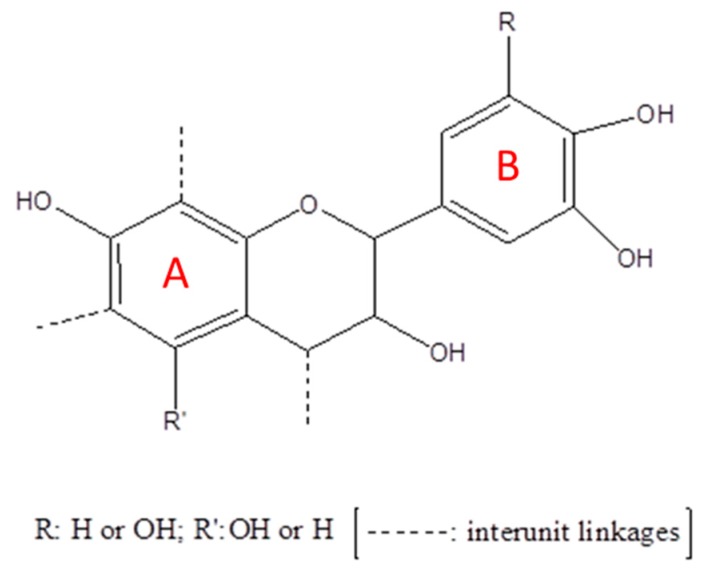
Chemical structure of tannins.

Prolonged exposure to ultraviolet (UV) radiation represents a well-known problem for polymeric materials [1,2,3]. It determines color loss, macroscopic fragmentation, and progressive reduction of molecular weight; hence, bettering understanding of the involved mechanisms and developing new methods to slow down this type of degradation are extremely active fields [4,5,6]. Modern additives for polymers are capable of absorbing the UV radiation or blocking the free radicals and peroxides produced during the degradation process [7,8]. These substances are generally produced from non-renewable sources, and bio-based materials should not rely on them to improve their performances [9,10,11]. On the contrary, molecules obtained from renewable resources are a desirable alternative [12]. Tannins, flavonoids, and other macromolecules classified as polyphenols are very interesting candidates from this point of view [13]. They are present in many types of plants and play a fundamental role in their protection against pests and diseases. In particular, tannins are one of the most abundant groups of compounds of vegetable origin and are readily available throughout the world [14]. Tannins can be classified as either hydrolyzable or condensed. Hydrolyzable tannins are made of simple phenolic products, being esters of gallic acid and their dimers (digallic acid, ellagic acid), and monosaccharides (especially glucose). They have already been used as partial substitutes for phenol in the manufacture of phenol formaldehyde resins [15,16]. Condensed tannins consist of flavonoid units with different degrees of polymerization. They have been associated with their precursors: catechins (flavans-3-ols), leucoanthocyanins (flavanes-3,4-diols) [17,18], and carbohydrates. This class of tannins is produced in the normal metabolism of plants, which explains why they are considered physiological and are widely present in plants across the planet [19,20,21]. Condensed tannins are used in many industrial applications (i.e., as glues for wood products, dyes for leather, etc.) [19,22,23] and constitute more than 90% of the world production of tannins. Due to its chemical structure (Figure 1), which constitutes a high amount of multiple phenolic hydroxyl groups, they could be used as a platform for producing reactive polyhydroxyl chemicals [24,25] and, hence, for precursors in polymer synthesis.

Among the several applications of tannins [26,27,28], their potential use as a protecting additive for polymers against the effect of UV rays has been proposed only in recent years. Samper et al. [29] reported the use of natural phenolic compounds derived from flavonoids, such as chrysin, quercetin, silibinin, and others, in the stabilization of polypropylene against thermo-oxidative degradation and UV radiation. The results show that these compounds provide the best performance in stabilizing against both oxidation and UV radiation.

Polyurethanes are among the most used polymers due to their wide range of properties. Their versatility allows the synthesis of different materials such as foams, coatings, adhesives, sealants, and elastomers. Applications can be found in fields like automotive industry, footwear, in construction as insulators, and, most recently, in medical devices. Polyurethanes are synthesized by reaction of an isocyanate, generally a polymeric isocyanate, with a polyol. As the synthesis of isocyanates is more complex than that of polyols, investigations usually focus on new bio-based polyols to reduce the PU carbon footprint while relying on synthetic isocyanates [30,31]. Various foams based on bio-based materials have been prepared and characterized in the last years [32,33,34]. Bio-based polyols offer a wide range of properties and, thus, the properties of the final material strongly depend on the polyol used [35,36]. The environmental sustainability [37,38] and the low cost render these materials environmentally friendly competitors of current synthetic polymers [39,40,41]. However, bio-based foams generally have lower resistance to environmental degradation than synthetic polyurethane [42]. In addition, almost no natural products are available as a substitute for synthetic UV stabilizers. In this respect, tannin can be employed as a reactive polyhydroxyl filler to prepare polyurethane composite foams; this is thanks to its chemical structure, alongside the aforementioned UV stabilizing properties. In fact, Ge et al. [43] reported that the phenolic hydroxyl groups present on the B-ring of tannin (Figure 1) are able to react with the isocyanatic group due to the higher electron densities on the oxygen atoms present in the B-ring compared to those in the A-ring.

In this work, the synthesis of polyurethane-based composite foams by using methylene diphenyl isocyanate (isocyanate source), ethoxylated cocoalkyl amine (polyol), and condensed tannins (as a UV stabilizer and additional –OH source) in the presence of a suitable amount of water (as a blowing agent), catalysts, and silicone surfactant was investigated. Different foam formulations were prepared to evaluate the effects of tannin and water contents on the foaming kinetics, mechanical properties, and foam morphology. The foams were exposed to accelerated degradation cycles, simulated by exposure to UV radiation, and the effects on the chemical and mechanical properties were evaluated.

## 2. Materials and Methods

### 2.1. Raw Materials

Methylene diphenylisocyanate (MDI) (Voranate M229 with isocyanate group (NCO) content equal to 31.1 wt % and functionality of 2.7) and ethoxylated cocoalkyl amine polyol (EtCO) (Lutensol® FA 12 with ethoxylated chains between 8 and 20 carbon atoms, with an average of 12) were purchased from Dow and BASF s.r.l. (Italy), respectively. CH_3_COOK and Niax PM40, chemicals used to regulate both the polymerization and blowing reactions, and L6164, used as a surfactant, were kindly provided by Momentive (Italy). Distilled water (H_2_O) was used as a blowing agent. Profisetinidin/prorobinetinidin condensed tannin (CT) was supplied by Silvateam S.p.a. (Italy). The moisture content of CT, assessed by a thermogravimetric method, was 2.5 wt % and was considered as part of the H_2_O blowing agent amount in the formulation of the composite polyurethane foams.

### 2.2. Preparations of PU and TaPU Foams and UV Treatment

The polyurethane foams were synthesized by using an OH(EtCO)/NCO(pMDI) ratio equal to 1. Different tannin based foam samples (TaPU) were obtained by properly changing the CT and distilled water contents with respect to the reference PU formulation. For sample preparation, CT, CH_3_COOK, Niax PM40, and L6164 were first mixed with EtCO at 200 rpm for 10 min using a magnetic stirring plate. Subsequently, distilled water was added to the mixture and mixed at 200 rpm for 1 min. Finally, MDI was added and stirred for 15 s. The resulting mixture was left to rise in a closed rectangular mold (10 cm × 10 cm × 3 cm) and, subsequently, the produced foams were cured at 40 °C for 5 h before any characterization. In order to minimize boundary effects and assure that the cellular structure was homogenous, chemical–physical and mechanical characterizations were performed on samples cut from the center of the plates. Foam samples were cut into cubic shapes with different dimensions. In Table 1, the analyzed formulations along with their sample codes are reported.

UV degradation tests were carried out by exposing samples to a 300 W UV lamp (Ultra Vitalux from OSRAM, Italy) in a closed box for selected times (3, 6, 12, or 24 h). Cubic samples (15 mm × 15 mm × 15 mm in size) were exposed at a distance of 20 cm from the lamp. The measured temperature and relative humidity in the box were 50 °C and 30%, respectively. In order to irradiate all surfaces, the sample was manually turned every 30 min.

### 2.3. Physical Properties Evaluaton

The foaming process was analyzed in detail by means of FOAMAT equipment (model 281 from Format, Messtchnik GmbH, Karlsruhe, Germany) and the software “FOAM” version 3.8 was used to analyze the recorded parameters. This device records changes in height, temperature, and dielectric polarization of the foam during its growth. The software also calculates several parameters such as induction, rise, gel, and curing times. Three samples for each formulation were tested.

The sample density was calculated as the ratio between the weight and volume of cubic specimens of about 30 mm × 30 mm × 30 mm. The weight was measured using an analytical balance (model AB265-S from Mettler Toledo L.L.C., Columbus, OH, USA), and sample dimensions were evaluated using a high-resolution caliper (model 500-181-30 from Mitutoyo, Japan). The calculated density values were averaged among four samples.

The mechanical behavior of foams in compression was measured by means of a universal testing machine (model 4304 from SANS, Shenzhen, China) with a calibrated 1 KN load cell. Parallelepiped samples were cut from foamed slabs 50 mm × 50 mm × 30 mm in size. Compression tests were carried out at room temperature and Young’s modulus and compression strength were calculated from compressive curves. The change in mechanical behavior during the UV treatment was evaluated on smaller samples by using a dynamic mechanical analyzer (DMA 2980, TA Instruments Inc., New Castle, DE, USA) in order to have a higher sensitivity with regard to the performance change. Specimens 10 mm × 10 mm × 10 mm in size were carefully cut to ensure a cubic shape with parallel surfaces. They were compressed at 25 °C and at a constant strain rate of 0.1 min^−1^ up to the maximum compressive stress allowed by the instrument (190 KPa).

### 2.4. Cellular Morphology and Spectroscopy

The morphology of the foams was analyzed with a scanning electron microscope (model S8000, Tescan Brno s.r.o., Brno, Czech Republic). Specimens were cut from the middle of the foam in the direction of growth. They were coated with gold with a sputter coater (model SC500, emScope-now Quorum Technologies Ltd, Laughton, UK) before observation. Low-magnification pictures of foam sections were taken with an optical microscope (model Z16 APO, Leica Microsystems GmbH, Wetzlar, Germany) and used to evaluate the mean cell size and the cell number per unit volume. Sections with at least 50 entire cells were chosen to assure a statistically representative evaluation.

FTIR spectra were recorded at room temperature by using a FT-IR spectrometer (model Frontier Dual Ranger, PerkinElmer Inc., Waltham, MA, USA) in attenuated total reflectance (ATR) mode from 400 to 4000 cm^−1^. ATR spectra were collected on the surface of the cubic foam sample before and after UV treatment. Spectra were recorded at 4 cm^−1^ resolution, and are the average of 64 scans. The spectral region ranging from 900 to 1800 cm^−1^ was normalized for an invariant peak at 1070 cm^−1^ and deconvoluted with OriginPro 8.0 software (OriginLab Corp., Northampton, MA, USA) by using Gauss–Lorentzian functions. The positions of absorption bands, corresponding to specific vibrational mode assignments of urethane linkages (related to coupled peaks of *ν_s_* C–N and *δ* N–H, and *ν_s_* C=O free and *H*-bonded) and urea linkage (related to the *ν_s_* C=O) [33], were determined by the automatic peak finding feature.

## 3. Results and Discussion

### 3.1. Foaming Process

**Figure 2:**
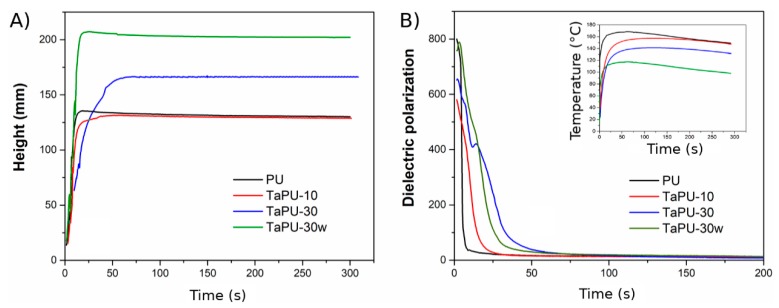
Kinetics curves of PU, TaPU-10, TaPU-30, and TaPU-30w foams: (**A**) height vs. time, (**B**) dielectric polarization vs. time (inset: Temperature vs. Time).

Selected polymerization kinetics curves (PU, TaPu-10, TaPU-30, TaPU-30w) are shown in Figure 2. Table 2 reports the induction time and the end of the rise time evaluated from the foaming kinetics detected by the Foamat device and reports the density of all samples. The induction time is defined as the time needed by the reacting mixture to change from clear to a creamy color [44], while the end of rise time represents the time needed by the foam to reach the maximum height [45]. These foaming parameters give an insight into how the reaction proceeds and how additives affect the foam formation. The blowing reaction of pristine PU starts almost immediately after the addition of MDI, while the presence of CT reduces the reaction kinetics. In particular, the induction time increases with increasing CT content. The presence of tannin increased the viscosity of the systems, in turn delaying the foaming reaction start in a similar way to that observed by Marcovich et al. [46].

The end rise time and maximum height also depended on the CT content. At CT contents of 10 wt % (Figure 2A, red curve) and 20 wt % (curve not shown), foams reached a height similar to that of the pristine PU foam (Figure 2A, black curve). Unlike for PU, a slowing down of the foam expansion rate was detected (see the end rise time values of the PU and TaPU-10 foams reported in Table 2). A peak was detected in the height curve of systems with quickly expanding formulations (such as PU and TaPU-30w in Figure 2). This is due to the slight collapsing (reduction in height) occurring when the maximum expansion degree is reached before the completion of the curing reaction of the polymeric matrix, which sets the polymer and quenches the cellular morphology. In the curves of polymeric systems having a slower expansion rate (TaPU-10 and TaPU-30 samples in Figure 2), the height curve did not show a peak before the end of the reaction.

At higher CT contents, the maximum height is higher than that of the pristine PU system, such as, for example, in the height vs. time curve of TaPU-30 (Figure 2A, blue curve). In fact, this system shows a delay in the foam setting time (see the end rise time in Table 2) and the absence of a peak in the height curve. The increase of the maximum height may be related to the increased viscosity with CT and to the nucleating effect of particles [47]. The water contained in the tannin powder is not readily available and this reduces the reaction kinetics, thus promoting the development of the blowing agent when the viscosity of the polymer is higher and reducing the gas escape towards the outside. Furthermore, CT particles act as cell nucleation sites and enhance the formation of supramolecular structures contributing to the increase of the expansion ratio and cell density [7]. These effects are not significant at low CT content, and pristine PU, TaPU-10, and TaPU-20 foams have similar density. Conversely, TaPU-30 and TaPU-40 foams exhibit a lower density with respect to the other systems (see Table 2).

The further addition of water to the formulation of PU and TaPU systems (Table 1) induced a faster blowing reaction (Figure 2A, green curve) due to the increased water molecules readily available for reacting with isocyanate [32] and a higher amount of volatile gas (CO_2_ and H_2_O). Consequently, the maximum height of the PU-w and TaPU-w foams significantly increased and the foam density decreased.

The dielectric polarization curves, reported in Figure 2B for selected samples, show the influence of CT on the reactivity of the polyurethane system [48] and the progress of the reactions occurring between functional groups as a function of time [49]. Dielectric polarization is essentially determined in chain-like molecules by the large dipole moments of their end groups (COOH, OH, NCO). The growth of the macromolecular chain during the crosslinking reaction ultimately suppresses the mobility of dipoles and their number; hence, it reduces the dielectric signal intensity. All the dielectric polarization curves show two distinct slope regions within a short time. The first region is related to the initial foam formation, while the second one is associated with the formation of crosslinks [33]. TaPU foams are characterized by a slow decrease of the dielectric polarization, indicating a lower reactivity of systems with CT with respect to the pristine PU foam. Such an effect becomes more evident as the CT content increases. The decrease of the reactivity with CT in TaPU foams is also in agreement with the decrease of the maximum reaction temperature during the foaming process (see inset of Figure 2B) and with the longer time to reach it. A similar effect was observed by Prociak et al. [50] for polyurethane foams based on other natural additives. The authors attribute this behavior to the fact that the addition of the filler increases the initial viscosity of the system, making the expansion of the reacting mixture more difficult [50]. The addition of water lowers the initial viscosity of the mixture and results in a more reactive system, also in the presence of CT, due to the higher amount of readily available water (see the green curve with respect to the blue one in Figure 2B). In fact, the dielectric polarization curve of TaPU-30 is crossed by that of TaPU-30w because the reaction is quicker and dielectric dipoles are more quickly depleted. After a long time, all the curves show a constant signal, proving that the reaction is complete.

### 3.2. FTIR Analysis

**Figure 3:**
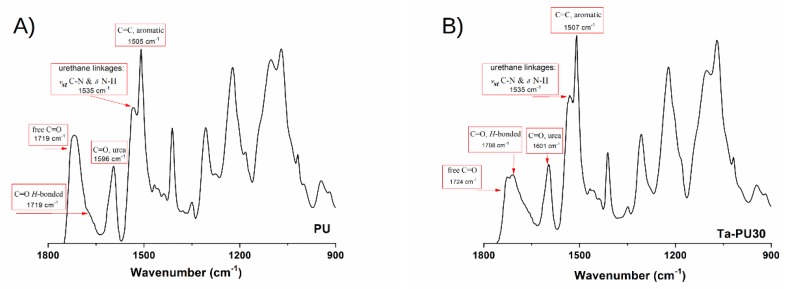
FTIR spectra of (**A**) pristine PU and (**B**) TaPU-30.

The FTIR analysis was performed on pristine PU and TaPU foams in order to assess the formation of urethane linkages. In Figure 3A,B, the FTIR spectra of pristine PU and TaPU-30, respectively, are reported. In both spectra, the absence of the band at 2230 cm^−1^ is due to the complete consumption of isocyanate groups, i.e., the complete reaction of the available reactants, in accordance with the result from Foamat measurements. Both spectra exhibit the characteristic vibration peaks of polyurethane foams; for instance, the carbonyl group stretching vibrations (free and hydrogen-bonded) related to the urethane and urea linkages, in the vibrational range 1600–1720 cm^−1^ [33,51]. Furthermore, the urethane domains were also detected through the vibrational modes, *ν_s_* C–N and *δ* N–H, at 1530 cm^−1^. The C–O stretching vibrations of EtCO segments were observed at 1200 cm^−1^.

Changes in the spectral response were recorded in presence of CT. Usually, H-bonding interactions shift the vibrational modes of participating functional groups (C=O, C–O, N–H, O–H, etc.) towards different wavenumbers (lower or higher) accompanied by a variation in the peak intensity. In the present case, the addition of CT induced a moderate shift towards higher wavenumbers of urea and urethane linkages (see Figure 3). This behavior is related to the increase of H interactions between the OH groups of CT and the CO and NR functional groups of EtCO. An increase in the C=O urea peak (at 1600 cm^−1^) and a corresponding reduction of C=O (free and H-bonded) of the urethane bonds was observed in the spectrum of TaPU-30 foam. This increase proves that CT catalyzes the formation of urea linkages, creating more stable H-linkages between the CT and the polyurethanic structure. The same behavior was observed for all the TaPU foams (data not reported for brevity). Additionally, an increase in intensity of the urea peak was observed in foams prepared with a larger amount of water.

### 3.3. Foam Morphology

**Figure 4:**
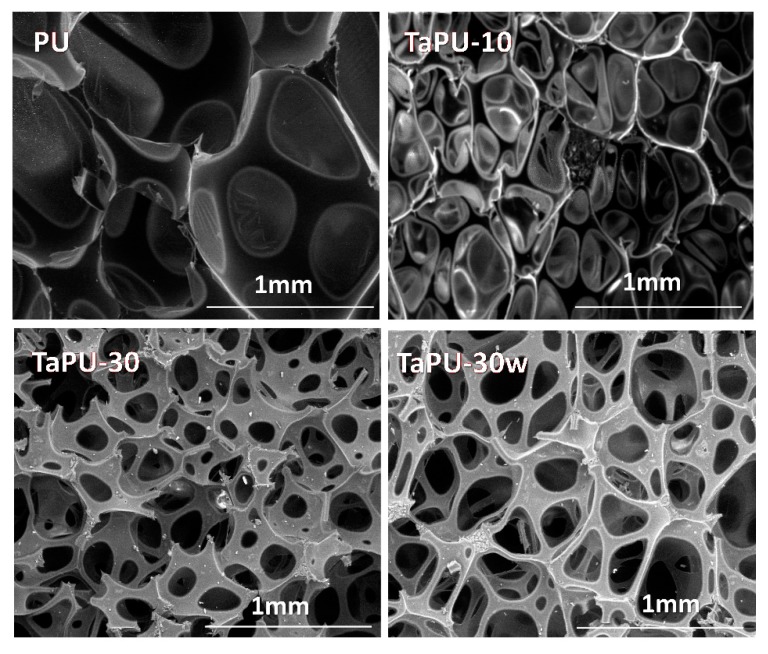
SEM images of PU, TaPU-10, TaPU-30, and TaPU-30w foams.

Figure 4 shows SEM images of selected foams. The foam morphology of the PU foam consists of closed cells with thin walls, while a very limited number of cells with broken walls was detected. With the addition of CT, the number of cells with broken or open walls increased. Foams prepared with a higher content of CT (such as TaPU-30) present a cellular structure with a high number of open cells. Alterations in the cell morphology are due to both the presence of CT, which in high amounts can induce wall rupture, and the increase of the urea concentration in the chemical structure, which can promote the opening of cell walls as also verified in polyurethane foams containing nanoclay and nanosilica [34,47]. The former effect could be more pronounced with increasing CT because of the large difference in hydrophilicity between tannin particles (hydrophilic) and the polymeric matrix (hydrophobic) and can also be promoted by the formation of particle aggregates. Pristine PU foams showed a low number of cells and a large mean cell size with respect to TaPU foams (Table 2). CT reduced the cell size and increased the number of cells; therefore, it can be concluded that CT has a nucleating effect. Despite this, the formation of tannin particle aggregates prevented a proportional increase of the number of nucleated cells with the CT content. At fixed CT content, the addition of water has the effect of increasing the mean cell size, since the higher availability of blowing agent can promote the cell growth at a constant number of nucleated cells (see SEM images of TaPU-30 and TaPU-30w in Figure 4).

### 3.4. Mechanical Behaviour

**Figure 5:**
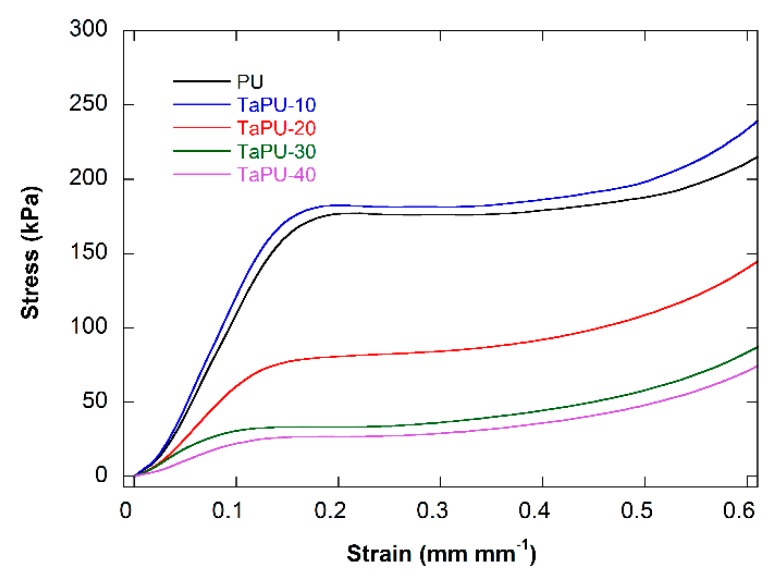
Stress/strain curves of PU and TaPU foams.

The effects of CT on the compressive stress/strain behavior of PU and TaPU foams are shown in Figure 5. Mean values of the mechanical parameters, in terms of Young’s modulus in compression, stress and strain at yield, and critical strain (defined as the strain at 190 kPa, from DMA tests), are reported in Table 3. Since the foams showed different densities, specific values of the mechanical parameters were calculated by normalizing to the foam density. The addition of CT had a non-linear effect on the mechanical behavior. At 10 wt %, CT improved both the modulus and yield strength, but with an increase in content, the entire curve lowered proportionally.

After taking into account the density, the TaPU-10 system still shows a higher stiffness with respect to pristine PU (+6%) and a similar compressive strength. A performance decrease with respect to PU was detected at higher CT content, and reductions by 42%, 63%, and 74% in compressive modulus and by 58%, 75%, and 76% in compressive strength were measured for the TaPU-20, TaPU-30, and TaPU-40 samples, respectively. In systems foamed with additional water, the trend was confirmed. The compressive modulus of TaPU-10w was increased by 12% and the compressive strength by 23% with respect to PUw, while reductions by 37%, 58%, and 72% in compressive modulus and by 2%, 31%, and 43% in compressive strength were measured for the TaPU-20w, TaPU-30w, and TaPU-40w samples, respectively. The reduction in compressive modulus and strength can be related to a) change in the morphological parameters (open cell content, degree of cell interconnections, wall and strut thickness) [52] and b) the presence of the CT aggregates, which were detected in edge sections in proportion with the CT content and which can limit their reinforcing effect.

### 3.5. UV-Treated Foams

**Figure 6:**
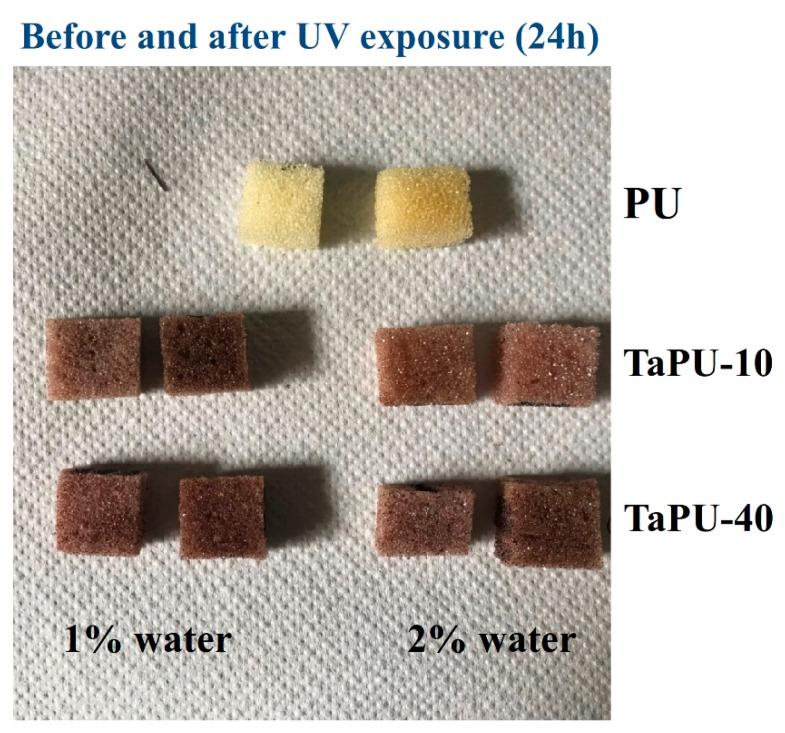
Images of PU, TaPU-10, and TaPU-30 foam samples before (left sample in the pair) and after (right sample in the pair) 24 h of UV exposure.

Exposure to UV rays in the range of 300–800 nm was performed to simulate weathering in outdoor light. In Figure 6, pictures of PU, TaPU-10, and TaPU-30 foam samples before and after 24 h’ exposure are shown to qualitatively show their appearance. The pristine PU foam turned yellow after 24 h as a result of oxidation reactions on the polymer backbone, and its surface became brittle. UV radiation modifies the chemical and physical characteristics of pristine PU, and a first distinguishable qualitative effect is the change in color of the exposed polyurethane matrix [53]. Several studies [54,55] have reported two mechanisms for photo-degradation in polyurethanes based on aromatic diisocyanate (MDI), namely, photo-oxidation of the aromatic functional groups and the direct photolytic cleavage of urethane groups. It has been demonstrated that the cleavage of the urethane group can result in a photo-Fries rearrangement [56]. It is important to note that CT can also act as a UV absorber in systems prepared with higher amount of water. In fact, although the tannin content per unit volume was reduced due to further expansion, the protective mechanism was still evident.

**Figure 7:**
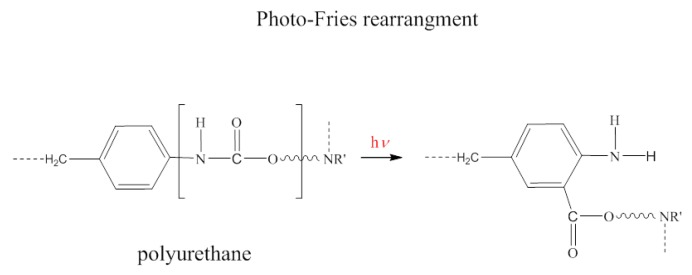
Photo-Fries rearrangement of polyurethane.

**Figure 8:**
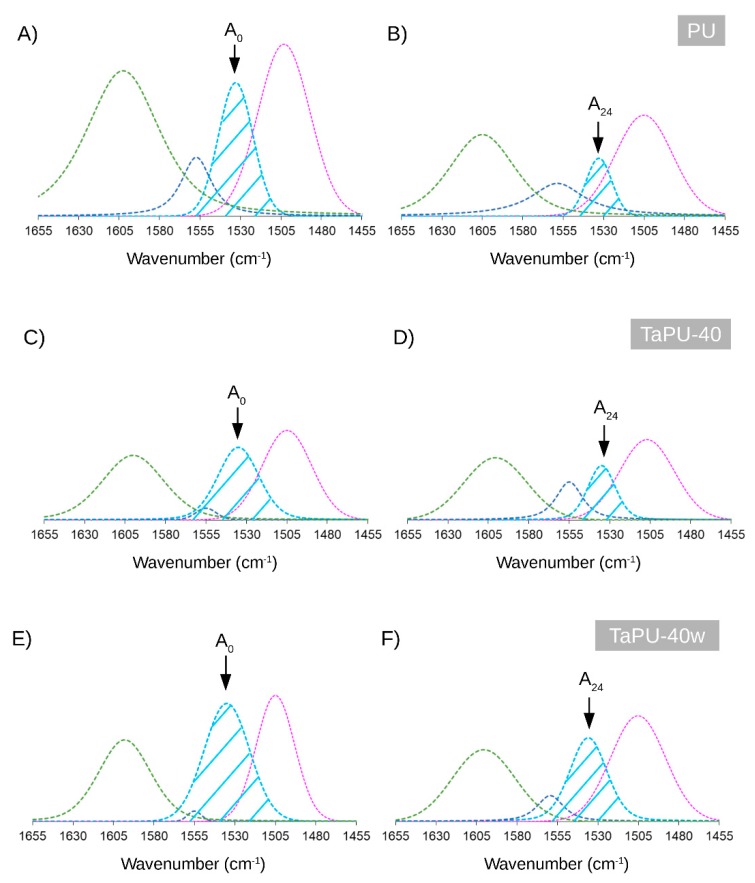
Deconvoluted spectra of PU, TaPU-40, and TaPU-40w before and after UV exposure for 24 h: (**A**,**C**,**E**) before UV treatment; (**B**,**D**,**F**) after UV treatment.

**Figure 9:**
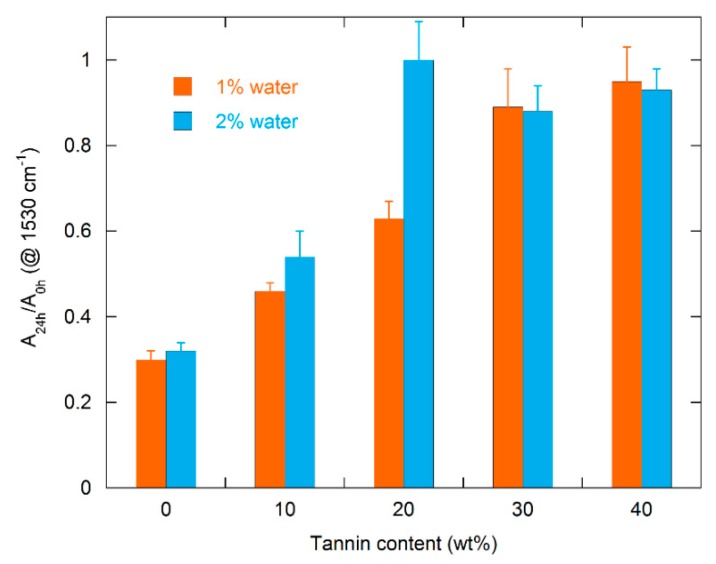
Ratio of areas under the 1530 cm^−1^ peak in FTIR spectra (A_24h_/A_0h_) measured before (A_0_) and after (A_24_) UV irradiation for 24 h.

As reported in several papers [32,33,51], chemical structure analysis by FTIR investigation helps to observe changes in the intensity of peaks related to the bonds that interact with the UV radiation. According to Rosu et al. [56], the urethane linkage, under the action of UV radiation, could undergo photo-Fries rearrangement (schematized in Figure 7) by inducing a reduction in the *ν_st_* C–N absorption peak area. The UV degradation effect can be estimated by calculating the area under the peak centered at 1530 cm^−1^ (related to *ν_st_* C–N). To quantify the UV degradation effect on PU and TaPU foams, the ratio (A_24h_/A_0_) between the area under the *ν_st_* C–N peak after the 24 h treatment (A_24h_) and its value before UV exposure (A_0_) was calculated from deconvoluted FTIR spectra. Selected FTIR spectra are reported in Figure 8 to show how the convolution process allowed us to distinguish the area under the 1530 cm^−1^ peak. The values of A_24h_/A_0_ are reported in Figure 9 for all samples. The pristine PU foam showed a low A_24h_/A_0_ ratio (32%) due to the occurrence of the photo-Fries rearrangement of urethane linkages. The presence of CT, on the contrary, reduced the sensitivity to UV degradation in all formulations. In particular, the highest protection of the urethane linkages—hence, the highest values of the A_24h_/A_0_ ratio—was obtained at 30 and 40 wt %. Thanks to the aromatic chemical structure, tannin behaves as a sacrificial UV inhibitor by absorbing UV radiation through the π → π* transition in un-saturated bonds. The degradation has an inverse relationship with the tannin content, and it is very low at 30 wt % and 40 wt %. The same behavior was detected in the lower-density foam series produced with added water.

**Figure 10:**
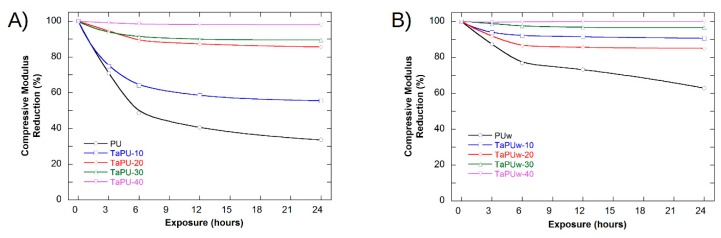
Percentage reduction of the compressive modulus as function of UV exposure time: (**A**) without added water; (**B**) with added water. (The solid lines are shown to guide the eyes.)

**Figure 11:**
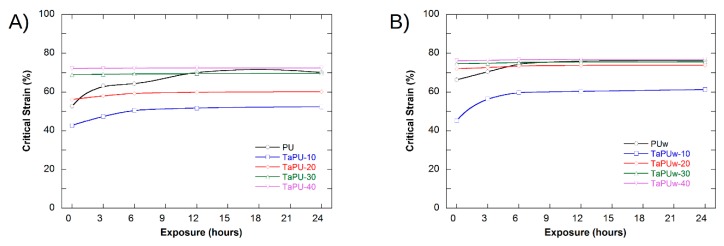
Variation of the critical strain as a function of UV exposure time: (**A**) without added water; (**B**) with added water. (The solid lines are shown to guide the eyes.)

The mechanical characterization of UV-treated samples, as well as of a not-treated foam used as a reference, was performed by setting the DMA to perform a static compressive test. The values of compressive modulus from DMA characterization are slightly different from the values from static tests, as expected, but the trend is in perfect agreement. The values of the compressive modulus for not-exposed (0 h) and exposed (3, 6, 12, 24 h) samples are reported in Table 4, while in Figure 10 and Figure 11 the changes in compressive modulus and critical strain are shown (variance of calculated values not shown). The compressive modulus decreased with the exposure time in all systems, while the critical strain increased. Pristine PU and TaPU-10 systems (Figure 10A) showed a strong change in the compressive modulus, and after just 3 h, reductions by 29% and 25%, respectively, were measured. In 6 h, the compressive modulus reductions reached 51% and 36%, respectively, but for longer times the change was limited (Figure 10A). The increase in critical strain followed an opposite pattern. It increased up to 53% and 42% for PU and TaPU-10, respectively, after 3 h of exposure (Figure 11A). At higher CT content, both the compressive modulus and critical strain showed very limited change. Therefore, the addition of tannin resulted in the attenuation of the degradation rate for both the compressive modulus and critical strain. The protective action of CT as a sacrificial inhibitor on the mechanical response of the foam is in agreement with the FTIR results, which did not show any significant change in the spectrum after UV exposure. Foamed systems produced with added water showed the same trend (Figure 10B and Figure 11B), but lower variation was detected. Such samples showed a lower sensitivity to the UV radiation with respect to the low-water-content series, and the compressive modulus reduction with the exposure time was further reduced and in excellent agreement with the corresponding results from the A_24h_/A_0h_ ratio reported in Figure 9. As also reported in the literature, the variation of the mechanical properties after UV exposure can be ascribed to the chemical–physical degradation of the polymer (oxidation, chain scissions, etc.) [57,58,59]. The reduction of the sensitivity to UV degradation is a clear demonstration that the addition of CT can be effective in protecting the polymer from UV weathering.

## 4. Conclusions

In this study, several polyurethane foams were synthesized by using methylene diphenyl isocyanate, ethoxylated cocoalkyl amine (polyol), and condensed tannin (CT). Water was used as a blowing agent in different amounts. The main findings of this study are as follows:

- The addition of CT to the formulation of the polyurethane foam affected the foaming process, reducing the reaction kinetics and lowering the foam density.
- The cellular morphology was improved by tannin, and more cells with a smaller size were generated.
- Increased water content in the formulation allowed a further reduction of the density, but the cell size was increased. Furthermore, water increased the amount of open cells.
- The mechanical performance of the foams was found to depend on the CT content but not in a linear way. In fact, CT had a reinforcing effect at 10 wt %, but in all other cases, tannin induced a significant reduction in both the compressive modulus and strength. These results are due to a) the increase in the open cell content and b) the presence of CT aggregates, detected in edge sections, that were in proportion with the CT content and limited the reinforcing effect.
- Foam stability against UV radiation is dependent on the tannin content. The FTIR analysis revealed a strong inhibiting effect of tannin on urethane linkage degradation during the UV treatment, particularly at high CT content. Thanks to its aromatic chemical structure, CT behaves as a sacrificial UV inhibitor because, as an aromatic compound with un-saturated bonds, it absorbs UV radiation through the promotion of π → π * transitions.
- The mechanical properties of the polyurethane foams were significantly affected by the UV exposure. CT strongly reduced the sensitivity to UV radiation, and at a content higher than 20%, the mechanical properties of such samples after UV treatment were almost unchanged.

## Figures and Tables

**Table 1:** PU and TaPU foam formulations.

Samples	EtCO/pMDI	Tannin (wt %) *	Water (wt %) **
PU	1	0	1
TaPU-10	1	10	1
TaPU-20	1	20	1
TaPU-30	1	30	1
TaPU-40	1	40	1
PUw	1	0	2
TaPU-10w	1	10	2
TaPU-20w	1	20	2
TaPU-30w	1	30	2
TaPU-40w	1	40	2

* wt % with respect to the total amount of polyurethane; ** wt % with respect to 100 parts of total polyol, TP.

**Table 2:** Characteristic times during the foaming process and the density of final foams.

Samples	Induction Time (s)	End of Rise Time (s)	Foam Density (kg/m^3^)	Mean Cell Size (μm)	Cells Number (×10^3^ cells/cm^3^)
PU	5	16	58.6 ± 2.3	1150	0.7
TaPU-10	12	43	61.5 ± 1.9	483	8.9
TaPU-20	13	52	60.6 ± 1.8	598	4.7
TaPU-30	20	68	51.1 ± 1.5	598	4.7
TaPU-40	23	70	51.1 ± 1.8	644	3.7
PUw	3	10	45.3 ± 2.1	1449	0.3
TaPU-10w	5	21	32.9 ± 1.3	920	1.3
TaPU-20w	4	20	38.4 ± 2.4	828	1.8
TaPU-30w	5	25	48.8 ± 1.4	644	3.7
TaPU-40w	6	26	49.6 ± 1.7	552	5.9

**Table 3:** Mechanical parameters from compressive tests on PU and TaPU foams.

Samples	Compressive Modulus (kPa)	Specific Modulus (kPa/kg/m^3^)	Compressive Strength (kPa)	Specific Strength (kPa/kg/m^3^)	Yield Strain (%)	Critical Strain (%)
PU	1475 ± 35	25.2	179 ± 12	3.1	19.2 ± 0.9	53 ± 3
TaPU-10	1640 ± 55	26.7	185 ± 11	3.0	18.5 ± 0.9	43 ± 3
TaPU-20	882 ± 21	14.6	78 ± 4	1.3	15.1 ± 0.4	56 ± 2
TaPU-30	481 ± 10	9.4	40 ± 3	0.8	18.3 ± 0.6	69 ± 2
TaPU-40	298 ± 9	6.6	33 ± 2	0.7	14.8 ± 0.5	72 ± 4
PUw	864 ± 19	19.1	45 ± 6	1.0	17.2 ± 0.8	66 ± 3
TaPU-10w	704 ± 21	21.4	41 ± 7	1.2	16.4 ± 0.5	45 ± 3
TaPU-20w	458 ± 13	11.9	39 ± 5	1.0	15.8 ± 0.8	72 ± 3
TaPU-30w	387 ± 11	7.9	34 ± 4	0.7	16.2 ± 0.4	75 ± 3
TaPU-40w	262 ± 6	5.3	28 ± 1	0.6	16.3 ± 0.6	76 ± 3

**Table 4:** Moduli of PU and TaPU foams at different UV exposure times.

Exposure Time (h)	Compressive Modulus (kPa)									
	PU	TaPU-10	TaPU-20	TaPU-30	TaPU-40	PUw	TaPUw-10	TaPUw-20	TaPUw-30	TaPUw-40
**0.0**	1080.6	1230.6	824.4	362.3	232.3	510.4	630.3	373.1	290.9	288.7
**3.0**	766.1	920.8	780.3	340.2	230.4	445.6	591.2	342.6	287.4	287.2
**6.0**	526.3	790.2	735.2	331.5	228.8	391.5	580.4	322.5	283.6	287.9
**12.0**	439.0	720.8	720.1	326.0	228.0	373.2	576.2	319.2	281.3	288.5
**24.0**	363.3	683.4	706.2	324.3	227.7	320.7	571.0	317.1	280.9	288.6
